# Nasal Valve: anatomy and physiology

**DOI:** 10.1016/S1808-8694(15)30795-3

**Published:** 2015-10-19

**Authors:** Carlos Eduardo Nazareth Nigro, Josiane Faria de Aguiar Nigro, Olavo Mion, João Ferreira Mello

**Affiliations:** 1 PhD - HCFMUSP, Assistant Professor of Otorhinolaryngology - Faculdade de Medicina de Taubaté, UNITAU; 2 PhD - HCFMUSP, Assistant Professor of Otorhinolaryngology - UNITAU; 3 PhD - HCFMUSP. Professor of Otorhinolaryngology - Faculdade de Medicina da Universidade de São Paulo; 4 Associate Professor of Otorhinolaryngology - University of São Paulo Medical School (FMUSP)

**Keywords:** anatomy, anatomy, nasal cavity, physiology, nose, acoustic rhinometry, nasal valve

## Abstract

The anterior portion of the nasal cavities, from the nostril to the nasal valve (NV), is the place of highest nasal resistance to airflow, paramount to nasal physiology. There are different terminologies for the same anatomic structures in the literature.

**Aim:**

The aim of this paper was to study the NV function and define clearly the structures of the anterior portion of the nasal cavities, mainly the region of the NV.

**Conclusion:**

Internum ostium is the anterior segment and isthmus nasi is the posterior segment of the NV region.

## INTRODUCTION

Valves are structures that regulate air or liquid flow within the human body. In the nose, the cartilage and the erectile tissue of the nasal cavities - especially those of the inferior nasal conchae and the nasal septum act as valves, regulating air flow.

The anterior portion of the nasal cavities, from the nostrils to the nasal valve (NV), is the region of the greatest nasal airflow resitance1 and where we have the narrowest segments of the nasal cavity^2^ thus, being very important for nasal physiology^3^^,^^4^ and the main nasal symptom - obstruction.

Subjectively, we can assess the NV using the Cottle test^5^^,^^6^. Anterior rhinoscopy is an objective way to evaluate the nasal cavity; however, the examiner's assessment of how much of the nasal cavity is obstructed or patent is subjective. Nasal endoscopy^7^, CT scan^8^ and MRI^9^ are described as tests capable to assess the nasal cavities, helping us in the diagnosis of anatomical variations associated with nasal disorders. Objectively speaking, rhinomanometry is a dynamic way to assess nasal cavity patency^10^ and nasal function; it aims at establishing nasal resistance, which is the difficulty of passing air through the nose, through the measurement of transnasal pressure and air flow. Acoustic rhinomanometry is a static way to assess nasal patency^11^ and geometry quantifying the areas of nostril cross-section all the way to the nasopharynx and nasal cavity volume between the two cross-sectional areas (CSA) chosen.

The literature has different terms used to define the same anatomic structures and, still, the same term used to define different anatomical structures and controversies regarding NV function. We carried out this study in order to review the literature regarding NV function and to better define anatomical structures in the anterior portion of the nasal cavities and their physiological importance.

## LITERATURE REVIEW

At the end of the XIX century, Mink^12^ is the one to suggest the concept of NV as the narrow opening formed between the caudal portion of the superior lateral cartilage (SLC) and the nasal septum, a 10 to 15° angle, as the region of maximum nasal resistance. In 1965, Van Dishoeck^13^ confirms this hypothesis from Mink and locates the NV in a coronal plane at the junction of the superior lateral and septal cartilages.

Bridger; Proctor14 use data of maximum inspiratory flow to conclude that the pyriform orifice region - where we have the head of the inferior nasal concha (INC), is where we have the greatest airflow resistance.

Bachman; Legler^2^ studied the cross-sectional areas of the nostrils, ostium internum and the pyriform orifice in plastic mold nostrils created from nasal cavities of live human beings, of 1.0; 1.4 and 0.7 cm^2^ respectively in other words, the narrowest point is the pyriform orifice.

Hirschberg et al.^1^, using a full body plethysmography reported that the area of greatest airflow resistance is located in the first two centimeters into the nasal cavity, being responsible for 56% of nasal resistance in basal conditions and by 88% after using topical decongestant.

Connel^15^ reports that in slow airflow situations, such as in the initial phase of inspiration, the air current has laminar flow and low energy expenditure; as its speed increases - because of irregularities on the nasal cavity surfaces - the flow becomes turbulent. With high nasal flow because of only one nostril being used or by paralysis of wing muscles after anesthetic block of the VII cranial nerve, there is SLC collapse^16^.

Jones et al.^3^ studied the effect of local anesthesia with lidocaine in the nasal vestibule floor on the perception of nasal airflow and nasal resistance reported by the patient, and concluded that thermoreceptors placed in the nasal vestibule contribute to the perception of nasal patency.

Shaida; Kenyon^17^ suggest that there are two valve mechanisms in the nose. The first, in the isthmus nasi - called internal valve in this paper - acts as a valve because of bulging of the INC head mucosa. The second is called external valve, and it is located in the vestibular border, because the inferior lateral cartilage (ILC) movement regulates airflow in the vestibule. In the normal nose, these two areas are connected by fibrous connections between the caudal border of the SLC and the cephalic border of the ILC.

Kelly et al.^18^ studied the airflow pattern of the nasal cavity and noticed that the NV region and the inferior airflow pathway are the regions where airflow reaches the highest velocity.

Cole^6^ divides the NV in its proximal and distal components. The proximal component, also called structural, is made up of the septal cartilage - which is rigid, and also by the inferior portion of the SLC, which has some mobility. Part of the distal component, also called functional, is the erectile tissue of the nasal septum and INC head.

Mlynski et al.^4^ studied airflow patterns in nasal models and concluded that the vestibule has the shape and acts as a tube joint, redirecting the air that comes from the front, the sides and below, thus creating a laminar flow. The isthmus nasi, in the inspiratory direction, has a concave shape that causes an airflow divergence towards the inside of the nasal cavity, having a similar effect to that of a concave optic lens on a light beam. In the conchae region, there is a reduction of turbulence and airflow velocity.

Lang et al.^19^ reported the presence of erectile tissue on the nasal cavity floor, in the pyriform orifice region, which contributes to airflow resistance in this site.

Huizing^20^ discusses the best terminology for the anatomical structures of the nasal cavity, stating that the ostium internum is the nasal cavity entrance by itself; the isthmus nasi is the narrowest place in the nasal cavity - synonymous with ostium internum; and suggests that the term NV should be used instead of ostium internum or isthmus nasi.

Morgan et al.^21^ carried out a study comparing nasal cavity geometry, by means of acoustic rhinomanometry, in normal adults - African-Americans, Caucasians and Orientals. They concluded that differences in cross-sectional areas (CSA) between Caucasians and Orientals happened because of a greater vascular component present in the nasal cavities of oriental individuals. In African descendants the pyriform orifice is wider, hence the apparent status of INC hypertrophy that we see in anterior rhinoscopy.

Grymer et al.^22^ define NV as the region between the ostium internum anteriorly all the way to the pyriform opening and the INC head posteriorly.

Lenders and Pirsig^23^ interpret the rhinogram obtained from the acoustic rhinomanometry test, indicating that the anterior nasal narrowing of the NV is the isthmus nasi.

Roithmann et al.^24^ used acoustic rhinomanometry noticed that the external nasal dilator is effective to treat patients with nasal obstruction caused by NV alterations.

On the base of the nasal pyramid we have the anterior nasal cavity openings, on the left and right, called nostrils. They are laterally bordered by the nasal wings - right and left, and medially by the columella. The columella corresponds to the mobile portion of the nasal septum, and it is a very important structure in establishing the nasolabial angle. The nostrils have a grossly oval shape, and in Caucasians its longest axis is the vertical one (leptorhine), in African-descendants it is the horizontal one (platyrhine) and, in other cases, its longest axis is oblique, having a roundish shape (mesorhine) ^25^. The ILC movement regulates airflow in the vestibule^17^.

The nasal vestibule is the entrance to the nasal cavity. It belongs to the nasal cavity; however, it is different from the rest of it, because most of its internal coating is skin. It has a medial wall made up of the septal cartilage and columella - which is formed by the junction between the medial crus of the ILC and its contralateral counterpart. Its lateral wall is concave and corresponds to the internal face of the ILC lateral crus, and it is covered by skin with hair and vibrissae. The literature is controversial in terms of nasal muscles function and anatomy^26^. The nasal muscles - nasal dilator muscle, nasal apex muscle, nasal muscle (wing part) and septum depressor muscle - according to the anatomical description (Chart 1) ^26^, have a dilatory function by voluntary contraction of the nostril and the nasal vestibule to increase air flow and in facial expression. Some authors^27^^,^^28^ state that there are mild involuntary contractions of these muscles, perceivable by electromyography during breathing, thus facilitating airflow, and also a passive movement caused by airflow itself. Individuals with NV obstruction caused by septum deviation and/or hypertrophy of the inferior turbinate head presented a greater electrical activity of the nasal muscles before corrective surgery^29^; a greater nasal muscle tone avoids cartilage collapse. Intranasal pressure seems to be more important than airflow to trigger the nerve signal to these muscles^30^. These muscles also have a stabilizing action due to the maintenance of nasal vestibule tonus that prevents its collapse^16^.

**Chart 1:** Origin and insertion of nasal muscles.

Name	Origin	Insertion
1. nasal m. cross sectional area	maxilla(lateral to the incisor fossa)	Nasal dorsum aponeurosis
2. nasal m., wing area	maxilla(incisor fossa)	Wing skin, accessory cartilage
3. nasal dilating m.	Inferior lateral cartilage	Wing skin (lateral crus)
4. nasal septum depressor m.	maxilla(incisor fossa)	Inferior lateral cartilage (medial crus)
5. nasal apex m.	Inferior lateral cartilage	Nasal tip skin (lateral crus)
6. procerus m.	occipitofrontal m.	Nasal dorsum aponeurosis
7. nasal wing and upper lip elevator m.	maxilla(frontal process)	Upper lip, nasolabial groove and nasal wing

Legend: m.: muscle.

The nasal vestibule has the shape of and acts as a tube joint, redirecting the air that comes from the front, below and the sides, thus creating a laminar flow^4^. It has thermoreceptors that contribute to the feel of nasal patency^3^. The increase in intranasal negative pressure causes ILC collapse in the nasal vestibule also helping to control airflow. In peripheral facial paralysis there is a reduction in nerve discharge to the nasal muscles, with consequent loss of muscle tone, causing the collapse of the nasal vestibule and of the NV, easily seen through nasal inspection. Posteriorly, we find an orifice, called ostium internum, which corresponds to the transition line between the skin squamous epithelium and the nasal mucosa - it is the anterior segment of the NV.

## DISCUSSION

The anterior portion of the nasal cavity, from the nostril to the pyriform opening is the narrowest portion of the nasal cavities, and alterations that reduce the lumen in this area are bound to have greater impact on nasal airflow when compared to more posterior regions^31^^,^^32^. A nose with the following characteristics has a higher risk of nasal obstruction feel: small size anteriorly, major differences between both sides, large decongestant effect on nasal permeability and symptoms of allergy and nasal infection^22^.

Shaida; Kenyon^17^ state that there are two valve regions: 1) the nostril and the vestibule; 2) the NV, is anteriorly made of the ostium internum and posteriorly by the isthmus nasi.

The 10 to 15° angle in the leptorhine, formed between the SLC and the nasal septum is called Mink's NV. The NV (Figure 1A, Figure 1B, Figure 2) is a tridimensional structure, anteriorly outlined by the ostium internum. Ostium internum, in Latin: inner door. Is a pear-shaped orifice, seen through anterior rhinoscopy that has the inferior border of the SLC as lateral border, the nasal septum as the medial one and the nasal cavity floor inferiorly, located at 1 to 1.5 cm from the nostril^33^. The tonus from the transversal portion of the nasal muscle stabilizes the SLC, avoiding its collapse. It is suggested that an indirect action of the nasal dilator muscle on the ILC would cause - because of the fibrous connections between the SLC caudal border and the ILC cephalic border 17 - SLC opening by translation, rotation and distortion^27^; however, how much this action would be effective to increase the ostium internum CSA, or if it would only stabilize such structure is a fact that deserves further studies, because there does not seem to have CSA changes to the NV during calm breathing^34^. During regular inspiration, the increase in flow velocity reduces the pressure that such flow has on the NV walls (Bernoulli principle); however, the rigidity of these structures prevents NV and nasal vestibule collapse.^16^ Farther posteriorly, the NV encompasses the isthmus nasi. Isthmus nasi is the Latin term meaning pathway to the nose. The isthmus nasi involves the pyriform orifice; nasal cavity floor which has erectile tissue^19^; the cavernous body of the nasal septum^35^; and INC head, which passes through the pyriform orifice at 0.3 cm in normal individuals and, after decongestion with topical nasal vasoconstriction agent (Figure 1B), it is in its threshold, reducing airflow resistance^1^. The quick volumetric reduction in the isthmus nasi's erectile tissue strongly suggests that the nasal cycle happens because of vasodilatation and vasoconstriction, and not because of edema caused by extravascular fluid buildup^36^. In African-descendants, the INC head is more visible through anterior rhinoscopy, since the pyriform orifice is wider, and therefore the INC head has greater importance regarding airflow resistance when compared to Caucasians and Orientals^21^. These structures form the second segment of the NV, called isthmus nasi, located at 1.65 to 2.65 cm in the nostril^33^. It is the place of lower CSA2 and greatest airflow resistance14 in the nasal cavity; mild changes to the CSA cause a major reduction in airflow^37^; any nasal obstruction in the NV or nasal vestibule results in greater negative intranasal pressure with a greater tendency towards collapse in these structures, and therefore greater nasal obstruction^38^. The isthmus nasi, in inspiratory direction has a concave shape, which allows for air current divergence to inside the nasal cavity; has a similar effect to that of a concave lens with a light beam^4^, the flow becomes turbulent^15^ and faster. The ostium internum and the isthmus nasi can act independently to increase resistance and reduce airflow. At the ostium internum, increasing the negative air pressure in the nasopharynx or inside the nose to increase airflow, there is a proportional increase in air pressure on the external walls of the nasal pyramid, since the intranasal pressure reduces all the way to a critical value when there is SLC collapse, reducing ostium internum CSA. In the isthmus nasi, changes to air quality (dry, cold or with impurities) cause vasodilatation to the erectile tissue. Thus, NV limits airflow to the nasal cavity to prevent it from exceeding the nose functional capacity regarding filtration, moisturizing and thermal regulation^14^.

**Figure 1A:**
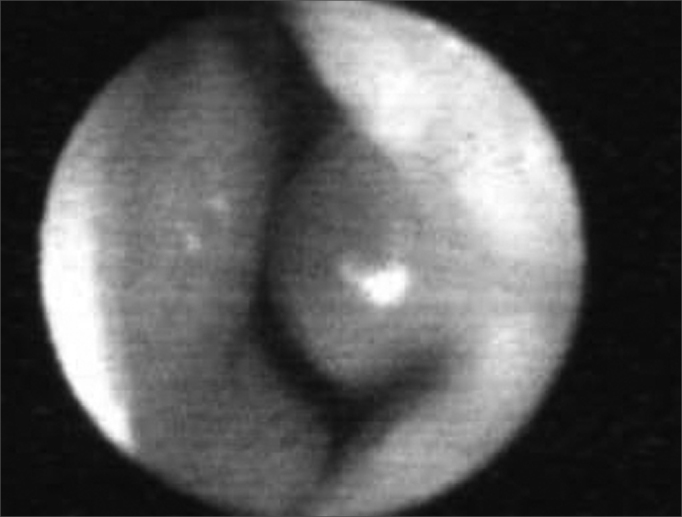
Baseline nasal valve photo.

**Figure 1B:**
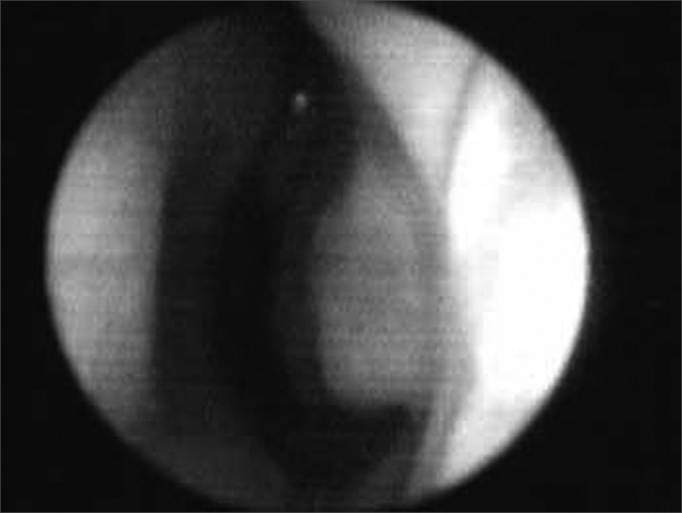
Nasal valve photo after using topical nasal vasoconstriction agent on the erectile tissue of the nasal cavity floor, the nasal septum cavernous body and the head of the inferior turbinate.

**Figure 2:**
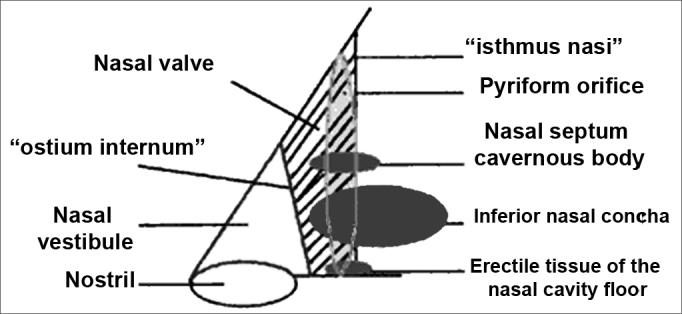
Nasal valve drawing.

Further studies could be carried out to check how much small involuntary contractions of the nasal muscles are effective to increase the nostril and nasal vestibule CSA and/or if this muscle electrical activity could have a predominantly stabilizing action during normal breathing.

The procerus and upper lip elevator muscles and nasal wing have a greater action on facial expression^26^.

### Nasal valve assessment

NV exam needs to be done carefully, since the very introduction of the nasal speculum in the nasal vestibule, as we normally visualize the nasal cavity itself, deforms the ostium internum. With the tip of the nasal speculum the nostril is opened, thus providing visualization of the vestibule and NV^6^. The endoscopic exam is also useful.

The Cottle test can be used to evaluate the NV. As we pull laterally the zygomatic region skin, we ask if the patient feels any improvement in his/her ipsilateral nasal patency; if yes, the test is positive. This maneuver changes the ostium internum's geometry. If there is any fibrosis in the ostium internum the test will be negative, thus a false-negative Cottle test.

**Figure 3:**
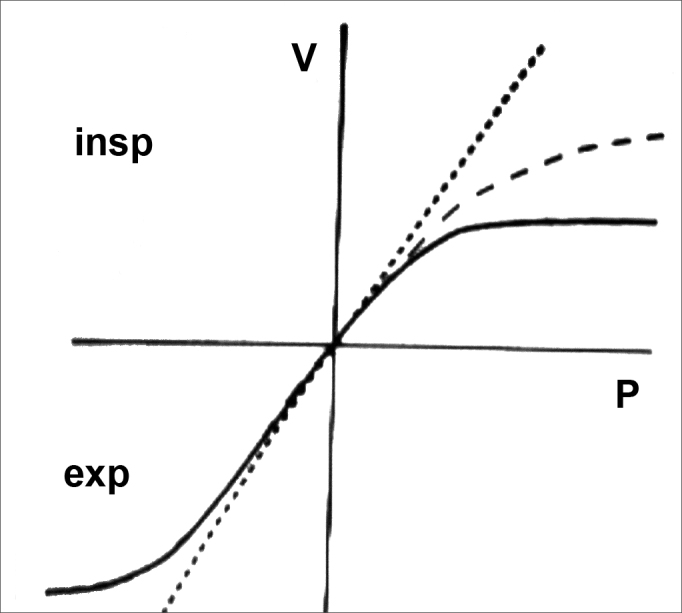
Rhinomanometry. Nasal valve typical curve.

Rhinomanometry presents a curve that is very typical of NV collapse with a reduction in airflow even with an increase in the negative pressure (Figure 3).

We can better assess NV components with the help of acoustic rhinomanometry, topical vasoconstrictor agent (VC) and external nasal dilator^24^.

Nigro et al.^39^ believe that in normal Caucasians, the second notch in the rhinogram represents the entire NV. Such notch starts at the ostium internum and its CSA represents the isthmus nasi area. After using the VC agent, the second notch moves anteriorly, and this happens because with a reduction in the NV's mucosal component present from the isthmus nasi, the ostium internum starts having a lower CSA than that of the isthmus nasi or the isthmus nasi CSA increases, but its CSA continues to be lower than that of the ostium internum^39^. The external nasal dilator used before and after the VC agent helps to differentiate. If there is a CSA increase again, this represents the ostium internum, since the external nasal dilator acts on the SLC. In preliminary studies we have seen that there can be a mild CSA increase which may represent the isthmus nasi; this mild increase happens because of the physical limitations associated to curve formation - the shorter NV narrowing makes the notch smaller. We need further studies to quantify these increases.

## FINAL REMARKS

Anatomical structures in the anterior portion of the nasal cavities are extremely important for nasal physiology. The literature is controversial as far as the nomenclature of NV structures is concerned. In the present paper we defined the NV as a tridimensional structure bordered anteriorly by the ostium internum and posteriorly by the isthmus nasi. The knowledge of the integrity of such structures and its differences help us better understand nasal patency and obstruction. Differences associated with ethnicity, gender and structural sizes - normal or abnormal, are extremely relevant for the clinical diagnosis of nasal obstruction. The correct diagnosis will lead to a successful clinical and surgical treatment, with special attention given to cosmetic surgical treatment.
